# Wilson disease in a Nigerian child: a case report

**DOI:** 10.1186/1752-1947-6-200

**Published:** 2012-07-16

**Authors:** Christopher Imokhuede Esezobor, Nora Banjoko, Adekunle Rotimi-Samuel, Foluso Ebun Afolabi Lesi

**Affiliations:** 1 Department of Paediatrics, College of Medicine, University of Lagos, PMB 12003, Lagos, Nigeria; 2 Department of Paediatrics, Lagos University Teaching Hospital, Idi-Araba, Mushin, Lagos, Nigeria; 3 Department of Ophthalmology, College of Medicine, University of Lagos, PMB 12003, Lagos, Nigeria

## Abstract

**Introduction:**

Wilson disease is rarely reported among African children. This report describes the second case report of a Nigerian child with Wilson disease in three decades.

**Case presentation:**

An eight-year-old African boy presented with generalized oedema and ascites and proteinuria. Over the next three weeks he developed conjugated hyperbilirubinaemia, severe coagulopathy and prominent extrapyramidal features consisting of rigidity, tremors at rest and in action, shuffling gait, slurred speech and emotional lability. Slit-lamp examination of his eyes revealed Kayser-Fleischer rings and sunflower cataracts. His serum caeruloplasmin level was 5mg/dL. Using the scoring system proposed by the 8^th^ International Meeting of Wilson Disease and Menkes Disease, a diagnosis of Wilson disease was made.

**Conclusions:**

Wilson disease does occur in African children, although the diagnosis is rarely made. A diagnosis of Wilson disease should be entertained in the evaluation of African children presenting with liver dysfunction and/or extrapyramidal neurological features.

## Introduction

Wilson Disease (WD) is a rare autosomal recessive disease resulting in a systemic overload of copper. The reported prevalence is 30 per million people [1]. However, apart from a report three decades ago, WD has not been described in children in Nigeria [2]. In this report we describe another Nigerian child with WD and highlight that, though uncommonly diagnosed in African children, WD should also be considered in the differential diagnosis of a clinical presentation of liver disease and/or extrapyramidal manifestation in children in Africa.

## Case presentation

An eight-year-old African boy, previously well, was referred to our unit on account of nephrotic syndrome. He had presented to our emergency department a day before with ascites, facial swelling and reduced urinary output in the preceding two weeks. After two days of hospitalization, he became deeply jaundiced with worsening of the generalized oedema. A day later, he had three brief episodes of generalized clonic seizures over a two-hour period and became increasingly drowsy. The seizures were aborted with diazepam and he remained seizure-free after a short course of phenobarbitone. A physical examination did not support a diagnosis of meningitis and other aspects of a neurological examination were normal. He had ascites and non-tender hepatomegaly; his spleen was not palpable. A review of his history did not reveal chronic or recent use of orthodox or non-orthodox medicines. In addition, there was no previous or current history of similar illness in his four siblings and close contacts. Both his parents were Africans, of the Igbo ethnic group from southeast Nigeria, but were not known to be blood relatives.

**Figure 1:**
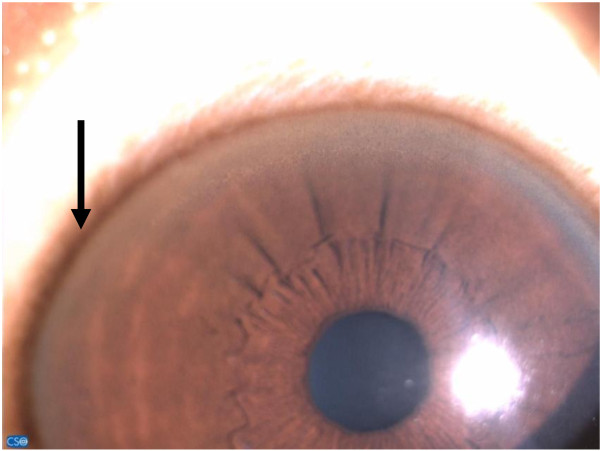
A slit-lamp examination showing Kayser-Fleischer ring (black arrow).

In the second week of hospitalization, our patient developed tremors of his hands while at rest and when reaching for objects. He became clumsy when performing chores involving the use of his hands. Subsequently, he was observed to be stiff globally, with his trunk arched forward and fisting of the hands (left more than right). His gait was noticed to be shuffling with a tendency to fall forward when trying to walk. At the same time, his face retained a wry smile and his speech became slurred and dysarthric. He frequently complained of generalized body pain and derived some relief when his clenched fist was helped open. He was also noticed to be emotionally labile; he cried inconsolably when asking for food. He was reviewed by a paediatric neurologist who pointed out the possible presence of Kayser-Fleischer (KF) rings on both eyes. A slit-lamp examination by an ophthalmologist promptly revealed the presence of both KF rings (Figure 1) and sunflower cataracts.

Initial investigations showed proteinuria of grade 1+ and a spot urine protein to creatinine ratio of 1. Table 1 shows the results for his liver function test during hospitalization. His serum electrolytes, urea and creatinine were within the normal reference range. An abdominal ultrasound scan revealed ascites and hepatomegaly with increased liver parenchymal echoes but no dilated intra- or extrahepatic ducts. However, his gall bladder was enlarged with a thickened wall and sludge within. Serology for human immunodeficiency virus, hepatitis B and hepatitis C viruses were negative. A serum sample for caeruloplasmin level was returned as having 5mg/dL of caeruloplasmin, using an immunoturbidimetric method (reference range: 25 to 45mg/dL).

**Table 1:** Results of liver function tests of the boy during hospitalization

**Tests**	**Values**	
	**First week**	**Second/third week**
Serum bilirubin: Total, Direct (mg/dL)	18.8, 14.0	0.5, 0.3
Serum protein (mg/dL)	5.3	6.3
Serum albumin (mg/dL)	3.4	1.1
Serum alanine transaminase (IU/L)	6	176
Serum aspartate transaminase (IU/L)	8	254
Serum alkaline phosphatase (IU/L)	24	258
Serum gamma-glutamyltransferase (IU/L)	40	49
Plasma thromboplastin: Test, Control (sec)	30.5, 82.0	30.5, 39.0
Prothrombin time: Test, Control (sec)	14.1, 85.3	14.1, 39.0
International normalized ratio	11.7	4.0

Using the scoring system proposed by the 8^th^ International Meeting of Wilson Disease and Menkes Disease [3], our patient achieved a score of 6 (compatible neuropsychiatric features = 2; K-F rings = 2 and caeruloplasmin level of 5mg/dL =2) and a diagnosis of WD was made.

His diet was restricted to high calorie, low protein and low copper meals. Vitamins, including vitamin K, and lactulose and neomycin were commenced. Control of the generalized oedema was achieved with furosemide and spironolactone. Long-term zinc and pyridoxine therapy was started. The use of trihexyphenidyl helped achieved some control of the spasticity and rigidity, sufficient to allow resumption of basic activities such as walking and holding objects. His liver dysfunction also improved, with near resolution of the international normalized ratio (from 12 to 4).

## Discussion

WD is a rare autosomal recessive disorder resulting in copper overload. Most cases have been reported in developed countries [4-6]. Apart from North Africa, where several cases of WD have been described, the diagnosis is rarely made in children and adults in Africa [7-9]. The higher frequency of detection in North Africa compared with the rest of Africa may be explained by the higher rate of consanguinity in North Africa, which makes an autosomal recessive disease more likely to occur [7].

Children with WD are usually normal at birth and may remain healthy for a variable period of time; most cases present in the second and third decade of life [4]. Our patient had conjugated hyperbilirubinaemia, ascites and severe dysfunction of the synthetic activity of his liver. Although he had proteinuria and generalized oedema, which informed the initial diagnosis of nephrotic syndrome, the proteinuria never reached nephrotic range and the progression of the oedema (ascites preceding pedal oedema) did not support a renal aetiopathogenesis for the generalized oedema. Indeed, it is now known that persons with WD could have some degree of proximal tubulopathy, which may be partial (as may be the case in our patient) or generalized (Fanconi syndrome) [10,11].

The neurological features of WD are primarily due to the deposition of copper in the lenticular nuclei, although areas like the brainstem and cerebellum can be affected [12]. In our patient, neurologic features developed within three weeks of hepatic presentation, and in the absence of the use of copper chelating drugs. He had most of the neurologic features described in the literature: rigidity, dystonia, dysarthria, tremor at rest, festinant gait and insomnia [1,12]. He was also emotionally labile with abrupt mood changes. Predominance of mood features in some patients explains the not uncommon presentation to psychiatrists [4]. In the presence of significant hepatic derangement, we viewed the seizures he had in the early phase of his hospitalization as part of the clinical features of hepatic encephalopathy.

We used the scoring system proposed by the 8^th^ International Meeting of Wilson Disease and Menkes Disease to make a definite diagnosis in our patient. A score of 4 or more makes the diagnosis of WD likely. This scoring system has been validated with high sensitivity and specificity as well as high positive and negative predictive values [6].

The long-term treatment of symptomatic cases of WD entails the chronic use of copper chelators and zinc, while liver transplantation provides a cure [1,5]. The copper chelators commonly used for WD are penicillamine and trientine hydrochloride but these chelators are not available in Nigeria. While we attempt to source trientine abroad, we based our decision to commence a zinc-based treatment on a case series which showed favourable improvement in symptomatic children treated with zinc only [13].

## Conclusion

We have described the second known case of WD in a Nigerian child, its rarity in the African population, and highlighted the varied clinical manifestations of the disease.

## Consent

Written informed consent was obtained from the legal guardian of the patient for publication of this case report and accompanying image. A copy of the written consent is available for review by the Editor-in-Chief of this journal.

## Competing interests

The authors declare that they have no competing interests.

## Authors’ contribution

CIE was the primary physician of the child and initiated the writing of the case. NB extracted the clinical data from the medical record. ARS performed the slit-lamp examination while FEAL made the provisional diagnosis of WD and was a major contributor to writing the case. All authors read and approved the final manuscript.
